# Influence of Cork Waste Processing in the Fabrication of Polymer Composites by Additive Manufacturing

**DOI:** 10.3390/polym17233167

**Published:** 2025-11-28

**Authors:** Alessandro Innocenti, Patricia Marzo Gago, Pedro Burgos Pintos, María de la Mata, Alberto Sanz de León, Sergio I. Molina

**Affiliations:** Dpto. Ciencia de los Materiales, Ingeniería Metalúrgica y Química Inorgánica, IMEYMAT, Facultad de Ciencias, Universidad de Cádiz, Campus Río San Pedro s/n, Puerto Real, 11510 Cádiz, Spain; alessandro.innocenti@uca.es (A.I.); patricia.marzo@uca.es (P.M.G.); pedro.burgos@uca.es (P.B.P.); maria.delamata@uca.es (M.d.l.M.); sergio.molina@uca.es (S.I.M.)

**Keywords:** cork, polymer composites, fused granular fabrication, large-format additive manufacturing, processing

## Abstract

This work evaluates the influence of processing on the production of various cork composites. Cork agro-waste with different particle sizes, namely fine cork (FC, D_p_ < 250 µm) and coarse cork (CC, 1 mm < D_p_ < 2 mm), was valorized. It was possible to process composites containing up to 20 wt.% FC and 15 wt.% CC using a twin-screw extruder. These composites were subsequently manufactured via large-format additive manufacturing (LFAM) using fused granular fabrication (FGF). The effects of cork concentration and processing duration in the extruder on particle integration, as well as on the mechanical, physical, and thermal properties of the composites, were studied. As expected, a linear decrease in mechanical properties was observed with increasing cork content. For the same cork content and longer processing durations, properties were similar for FC and CC composites. Shorter processing durations in the extruder minimized degradation of FC. However, partial degradation occurred during FGF printing, leading to the formation of composite foams with increased porosity, lower density, and enhanced thermal insulation.

## 1. Introduction

Cork is a natural material obtained from the bark of the Quercus suber oak, which grows mainly in the western Mediterranean region, particularly in Portugal, Spain, and some areas of Italy and France. It possesses a unique combination of properties, such as high flexibility and resilience, excellent acoustic and thermal insulation, a near-zero Poisson ratio, and remarkable fire resistance [1,2]. Every year, thousands of tons of cork are produced, most of which is used for bottle stoppers and as insulation panels for the construction industry. It is estimated that around 70% of global cork production is devoted to bottle stoppers, corresponding to over 12 billion corked bottles per year [3,4]. However, during stopper manufacturing, up to 30% of the starting material is lost as waste, which is usually burned without any further valorization. Moreover, used cork stoppers rarely have a second life and are typically discarded in landfills or incinerated for energy recovery [5]. In recent years, growing environmental awareness has prompted efforts to find sustainable alternatives for reusing both cork powder and recycled stoppers within a circular economy framework, particularly in the regions where cork production is concentrated [6,7,8].

In this context, several researchers have developed polymer-based composites incorporating cork as a filler. The inclusion of cork not only reduces the proportion of petroleum-derived plastic but also reuses a renewable bio-based material, yielding a new value-added product. Cork composites have been prepared using polymer matrices such as polypropylene (PP) [9] or polyethylene (PE) [10], as well as biopolymers like polylactic acid (PLA) [11], chitin [12], or bio-based polyurethane [13]. Most of these materials are produced by conventional processing techniques such as injection or compression molding [14,15].

In recent years, additive manufacturing (AM) technologies have revolutionized the manufacturing sector by enabling the fabrication of parts directly from digital models through a layer-by-layer approach [16]. Among the different AM techniques, material extrusion processes—including fused filament fabrication (FFF) and fused granular fabrication (FGF)—are particularly relevant for polymer composites. FGF is especially attractive because it can be easily implemented in large-format additive manufacturing (LFAM), allowing the production of parts exceeding 1 m^3^ in volume. This approach has found increasing interest in industrial sectors such as construction, automotive, and transportation [17,18,19,20,21]. The use of large-diameter nozzles in FGF enables the incorporation of higher filler loadings and larger particles or fiber sizes without clogging issues, facilitating the development of composites with enhanced structural and functional properties [22].

The use of cork in AM has already been explored by several authors. Studies have reported successful printing of cork composites based on PLA [23], thermoplastic polyurethane (TPU) [24], polymethyl methacrylate (PMMA) [25], acrylonitrile-butadiene-styrene (ABS) [26], styrene–ethylene–butylene–styrene (SEBS) [27], and polyamide (PA) [28]. However, one of the major challenges in these systems is the poor interfacial compatibility between the hydrophilic nature of cork and the typically hydrophobic polymer matrices. This incompatibility promotes cork particle aggregation during processing, leading to embrittlement of the composite. Several strategies have been proposed to overcome this limitation, including surface modification of cork particles to improve interfacial adhesion, or the use of solvent-assisted compounding methods such as solvent casting [25,29]. While these approaches can enhance compatibility, they often present drawbacks related to scalability and the use of environmentally harmful organic solvents. The most practical and industrially viable alternative remains melt compounding using a twin-screw extruder. Nevertheless, this process can partially degrade cork due to thermal and shear stresses and also induce densification of its cellular structure [10,30]. As a result, the material loses its characteristic lightweight and insulating properties, which limits its functional contribution to the final composite [31].

Although many studies have demonstrated the feasibility of processing cork composites by melt extrusion, only a few have succeeded in preventing cork densification [32]. Furthermore, there is still a lack of systematic analyses on how processing conditions influence the structural and functional properties of cork-based composites. Comparisons among studies are further complicated by differences in particle size distributions and in the sources of cork used. Therefore, this work investigates the effect of twin-screw extrusion processing on the structure and properties of cork-filled composites using acrylonitrile–styrene–acrylate (ASA) as the polymer matrix. Different cork particle sizes and filler loadings are evaluated to identify the processing limits of these materials. The resulting compounds are subsequently used to fabricate parts through fused granular fabrication (FGF), and the influence of processing on their mechanical, physical, and thermal behavior is comprehensively assessed. This study provides new insights into the development of sustainable, cork-based composites suitable for large-format additive manufacturing, contributing to the valorization of cork residues within a circular and resource-efficient production model.

## 2. Materials and Methods

### 2.1. Materials 

ASA pellets (general purpose ASA for injection molding) were purchased from LG Chem (Seoul, South Korea). Cork powder form recycled cork stoppers was supplied by Catalan Cork Institute (ICSURO) (Girona, Spain). Two different particles sizes were used in this work: fine cork (FC) with a particle size D_p_ below 250 µm and coarse cork (CC) with an average size of 1 < D_p_ < 2 mm.

### 2.2. Compounding of Cork Composites 

**Figure 1:**
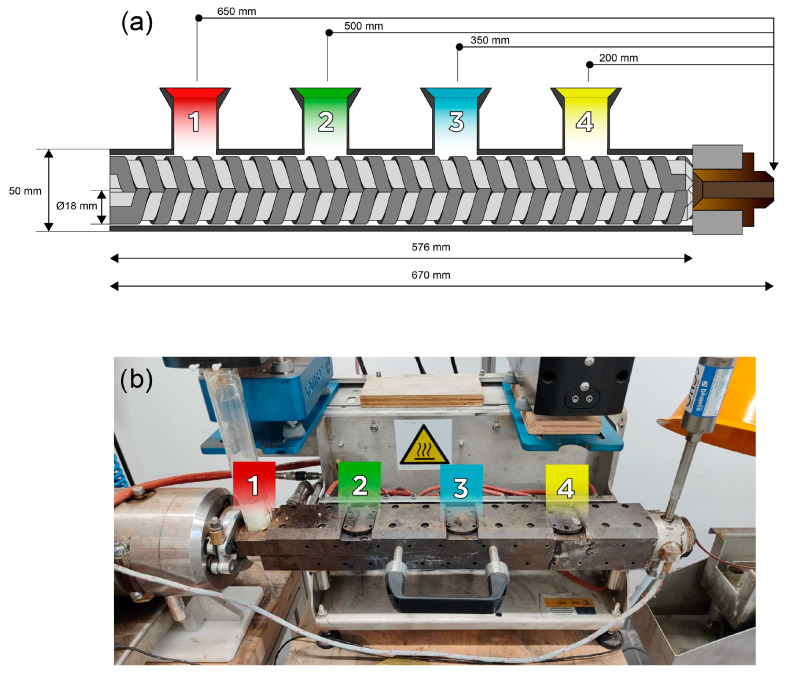
(**a**) Scheme and (**b**) digital picture of the twin-screw extruder used in this work.

Cork composites were synthesized in a twin-screw extruder, Scamex Rheoscam D20 (Isques, France), with an 18 mm screw diameter, an L:D of 32 and a screw speed of 100 rpm. Different cork concentrations from 5 to 20 wt.% were tested and different inlets of the extruder were tested to study the influence of processing on the structure and properties of the composites. Figure 1 shows a simplified scheme of the extruder used, with 4 inlets and 5 heating zones. A summary of the composites prepared with FC and CC are presented in Table 1 and Table 2, respectively. A temperature profile of 245-250-250-250-245 °C was used for all the composites for which ASA was introduced in inlet 1, while a temperature profile of 90-220-245-250-245 °C was used for all the composites for which ASA was introduced in inlet 2. In cases that turned out successful, a continuous filament was obtained and cut into small pieces measuring a length of ca. 2 mm using a Scamex pelletizer (Isques, France).

### 2.3. Fabrication via FGF LFAM and Postprocessing 

The synthesized cork composites were then manufactured by fused granular fabrication using a Discovery 3D Granza large-format additive manufacturing printer purchased from Bárcenas CNC (Ciudad Real, Spain). Two types of plates were printed in the XY and XZ directions, according to ISO 17295. Both plates were printed using a bead width of 2 mm, layer height of 1 mm, and printing speeds of 50 mm/s for the XY plates and 25 mm/s for the XZ plates. A temperature profile of 215/220/225 °C, optimized in previous work, was used in all cases [31]. These temperatures correspond to the three heating zones of the extruder, the last one being the closest to the nozzle. In all cases, the platform temperature was set to 100 °C to ensure good adhesion of the first layer [31,33]. Then, a LEKN(C1) 3020 CNC Router Machine Kit CNC (Ciudad Real, Spain) was used to cut the specimens for tensile testing and to allow for thermal conductivity out of the printed plates, according to ISO 527 and ISO 22007 standards, respectively. A 3 mm diameter flat milling cutter with two cutting edges was used to machine the specimens at a speed of 5000 rpm and a feed rate of 200 mm/min.

### 2.4. Characterization

The melt flow rate (MFR) was measured using a Lonroy LR-A001-A machine (Dongguan, China). The measurements were obtained every 50 s while applying a load of 10 kg at 225 °C for all the materials used in this work. At least 3 independent measurements were performed to ensure the reproducibility of the results. The density of the printed parts was measured in three independent samples using a Dahometer DH300 (Guangzhou, China). The mechanical characterization of the printed specimens was assessed by tensile testing in a Shimadzu AGS-X machine (Kyoto, Japan) using a constant speed of 1 mm/s. At least 5 specimens of each material were tested in all cases, in agreement with ISO 527. The fracture surface of the XY tensile-tested specimens were examined by scanning electron microscopy (SEM) on an FEI Nova NanoSEM 450 microscope (Hillsboro, OR, USA) equipped with a field emission gun. Specimens were previously coated with a 10 nm Au layer in Balzers SCD 004 Sputter Coater (Schaumburg, IL, USA). The thermal stability of the materials was examined by thermogravimetric analysis (TGA) in Q50 (TA Instruments, New Castle, DE, USA). Following a typical procedure, a temperature sweep from room temperature to 600 °C was performed using a constant rate of 10 °C/min. All the TGA experiments were carried out under a constant nitrogen flow. The thermal conductivity of ASA, 11FC_15, 14FC_15 and 11CC_15 was measured using a DTC-25 conductivity meter (TA Instruments, New Castle, DE, USA) in accordance with the ASTM E1530standard. Analysis of variance (ANOVA) with a significance level of α = 0.05 and Tukey’s tests were conducted to determine the significance of the mechanical properties obtained from the tensile tests, and the density and thermal conductivity results.

## 3. Results and Discussion

Different composites were produced using FC and CC at concentrations ranging from 5 to 20 wt.% by introducing ASA and cork particles through different feed ports to evaluate the influence of particle loading and residence time on composite formation. Prior to processing, both cork and ASA were dried as described in the previous section. Table 1 and Table 2 summarize all composites prepared with FC and CC, respectively.

In all cases, cork composites containing up to 15 wt.% of either FC or CC were successfully manufactured when using inlets 1 and 2. A composite containing 20 wt.% FC could also be produced through inlet 1, likely because the smaller particle size of FC and the longer residence time at this inlet facilitated improved homogenization along the screws, resulting in a uniform material at the extruder outlet. Attempts to increase the cork content beyond these limits produced filaments that were too brittle to be wound and pelletized, enabling us to determine the maximum cork concentration processable with this twin-screw extruder. It was not possible to use inlets 3 and 4 for ASA feeding, as the polymer could not be adequately introduced and caused blockages. For this reason, cork could not be added through a different inlet than ASA, except in the specific case where FC was added through inlet 4 while ASA was added through inlet 1. This configuration reduced the average residence time of FC inside the extruder to approximately 40 s, compared with the already short residence times measured for inlets 1 (ca. 120 s) and 2 (ca. 90 s), and therefore further minimized cork degradation, which remained limited in all cases due to the brief processing times involved.

**Figure 2:**
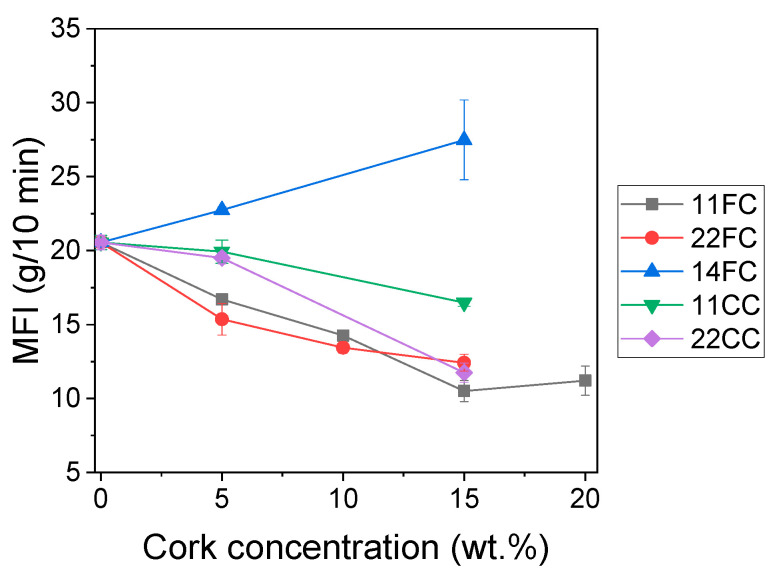
MFI of FC and CC composites.

Then, a study of the composites’ flowability was conducted to better understand whether the presence of cork particles impacted melt viscosity, limiting the processability of the material by FGF and affecting the mechanical properties of the printed objects. Figure 2 shows that for both FC and CC composites, the melt flow index (MFI) decreased with increasing cork concentration, regardless of whether it was introduced at inlet 1 or 2 (composites labeled as 11FC, 11CC, 22FC and 22CC). This effect is expected because as the cork content increases, the mobility of the ASA polymer chains is limited, and melt viscosity decreases due to steric hindrance effects. However, in the case of 14_FC composites, the MFI increased above that of ASA, which was unexpected because, as mentioned above, an increase in particles typically decreases melt flowability. We believe this may have been because, by introducing cork at an extruder inlet so close to the outlet, its degradation was minimized during the compounding process. This potentially led to the cork degrading during the MFI measurements, carried out at 225 °C. Under these conditions, the cork degraded inside the equipment, releasing gases that resulted in a lower-than-expected apparent MFI. Since the MFI process was carried out under conditions like those used in FGF printing, it was expected that this gas release would also occur during printing. In any case, all the MFI values obtained are between 10 and 40 g/10 min for all the materials tested, which is considered valid for FGF [34], implying that they can all be processed by this technology.

**Figure 3:**
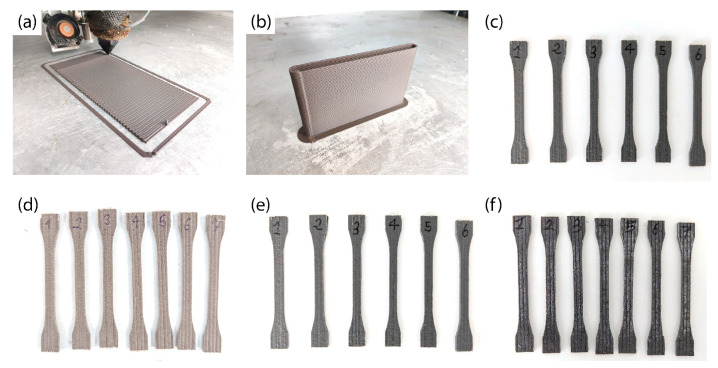
Digital pictures of (**a**) an XY plate using 11FC_10 and of (**b**) an XZ plate using 11FC_10, (**c**) 11FC_15, (**d**) 14FC_15, (**e**) 11CC_15, and (**f**) 22CC_15 tensile testing specimens manufactured by FGF. All the images were taken under the same light and exposure conditions, so they are directly comparable.

Then, the cork composites were printed by FGF using a pre-optimized temperature profile, which ensured that the cork composites could be printed with minimal degradation [31]. It is important to note that, although some studies report that certain components of cork begin to degrade at temperatures above 200 °C, these degradative changes do not have a significant impact on the mechanical properties of cork until it is exposed to temperatures exceeding 250 °C. Therefore, under typical processing conditions, cork can be considered mechanically stable [29,35]. All composites were printed using the same temperature profile and printing conditions to ensure comparable results, regardless of the amount and size of cork. Deviations between the designed and actual dimensions were very low, with surface roughness in the order of tenths of a millimeter, in line with typical LFAM tolerances reported in the literature [36]. Then, the tensile specimens were obtained via CNC machining, and their dimensions were measured in all cases to ensure compliance with ISO 527 standards, guaranteeing their dimensional accuracy. Taking this into consideration, in all cases, the composites were obtained with a good degree of homogeneity in both the dimensions of the deposited material and its color (Figure 3). Interestingly, the 14FC printed samples exhibited a lighter brown color (see for instance 14FC_15 in Figure 3d), demonstrating that it was possible to decrease cork degradation when it was introduced in inlet 4.

**Figure 4:**
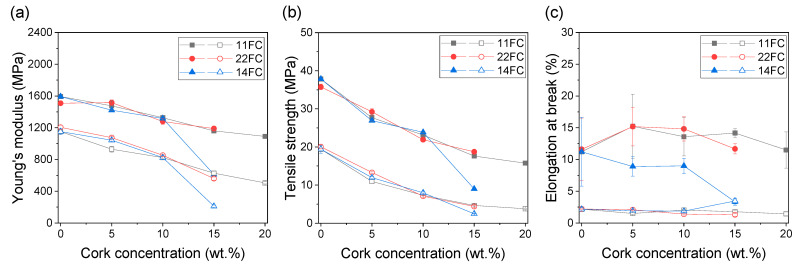
(**a**) Young’s modulus, (**b**) tensile strength, and (**c**) elongation at break values of 11FC (black), 22FC (red) and 14FC (blue) composites as a function of the cork concentration. Filled symbols correspond to XY tensile specimens while hollow symbols correspond to XZ tensile specimens.

**Figure 5:**
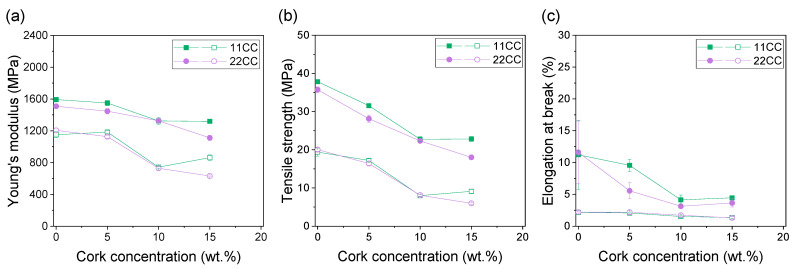
(**a**) Young’s modulus, (**b**) tensile strength, and (**c**) elongation at break values of 11FC (green) and 22FC (purple) composites as a function of the cork concentration. Filled symbols correspond to XY tensile specimens while hollow symbols correspond to XZ tensile specimens.

Next, the mechanical properties of the composites were tested. Figure 4 and Figure 5 show the mechanical properties (Young’s modulus, tensile strength and elongation at break) extracted from the different tensile curves for all composites prepared by FGF with FC and CC composites, respectively. In both cases, the results for XY (filled symbols) and XZ (hollow symbols) specimens are illustrated, which represent the actual mechanical properties of the printed material and the interlayer adhesion due to the intrinsic layer-by-layer process of FGF technology, respectively. Figure 4 shows that, in all cases, the differences between composites 11FC and 22FC are practically negligible, regardless of the cork content. ANOVA analysis shows only significant differences for Young’s modulus between 11FC_5 and 22FC_5 XZ specimens and the elongation at break value between 11FC_15 and 22FC_15 XY specimens. This is probably because the difference in residence time between inputs 1 and 2 was very small; therefore, it did not substantially modify the integration of the cork in the ASA matrix, giving rise to virtually identical mechanical properties. This also occurred in a similar way for composites 11FC and 14FC, where the more statistically significant differences could be observed in sample 14FC_15. In all cases, a linear decrease in Young’s modulus and tensile strength was observed for both the XY and XZ specimens as the cork content increased. The elongation at break values, however, remained constant, without showing significant variations in general with respect to pure ASA, except in the case of 14FC composites, where a marked decrease was observed as the cork content was increased. Similar behavior can be observed in Figure 5 for the CC composites, where composites 11CC and 22CC also had decreased general mechanical properties as the cork content increased. In this case, a slight improvement was observed in the 11CC composites (for instance, results of the ANOVA show that there are statistical differences in the mechanical properties of 11CC_5, 22CC_5, 11CC_15 and 22CC_15 XY specimens), which may be have been related to the higher MFI, as previously observed in Figure 2, leading to the better diffusion of the polymer chains between and within the printed layers. Furthermore, the values observed for Young’s modulus and tensile strength are very similar when comparing FC and CC composites, demonstrating that particle size has a small influence on these mechanical properties. In particular, no statistical differences were observed between 22CC and either 11FC or 22FC composites for all the XY specimens studied in terms of Young’s modulus or tensile strength. The elongation at break values, however, were lower in the case of CC composites, probably because they favored the appearance of fracture initiation points that would have embrittled the composite, being larger in size. In general, the reduction in mechanical properties in these composites was an expected effect, since cork does not play a role as structural reinforcement, given that it has worse mechanical properties than the ASA used as a matrix; this has been observed before in previous studies with cork [31,37,38] and other examples of agro-waste [39,40,41].

**Figure 6:**
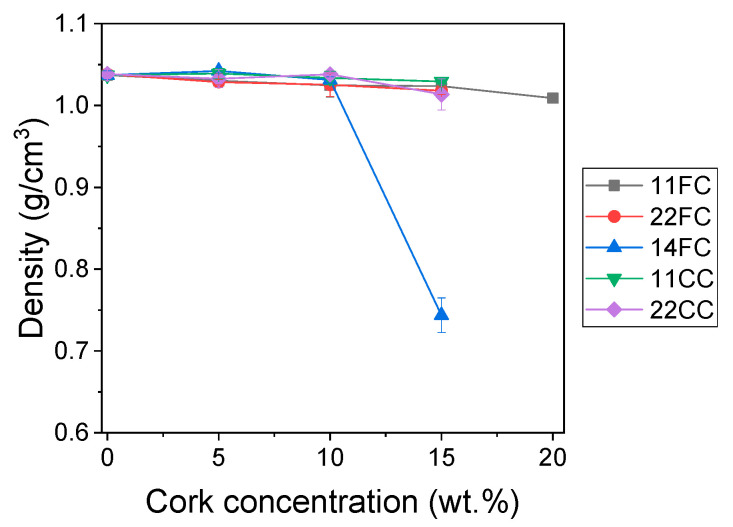
Density of the FC and CC composites after manufacturing by FGF.

Then, the density of the printed objects was measured (Figure 6) and it was observed that there were no significant differences between the cork composites and ASA (around 1.04 g/cm^3^) in all cases except for 14FC_15, which had a density of ca. 0.75 g/cm^3^. This unusual behavior is in agreement with the MFI results, where the higher cork content introduced in inlet 4 prevented degradation during compounding but led to partial degradation during the FGF printing process, likely releasing gases that could become trapped in the composite structure, resulting in a lower apparent density. For the remaining composites, the density values are higher than expected, since cork has a nominal density of 0.2 g/cm^3^. However, the values observed in these composites are slightly below the value provided by the ASA supplier (1.11 g/cm^3^). This small decrease is probably due to the gaps generated between strands and layers deposited during the FGF process itself, according to previous reports [29,42], not due to the cork. Hence, the influence of the cork particles on the density of the composite was not observed, probably because they were crushed during the extrusion process, as previously observed [31,43]. This would have made the cork lose its characteristic cellular structure, which is what makes cork a lightweight material. Other studies show that it is important to further minimize the impact of extrusion by modifying the cork particles on the surface or using alternative methods such as solvent casting to obtain lightweight cork composites [25,29].

**Figure 7:**
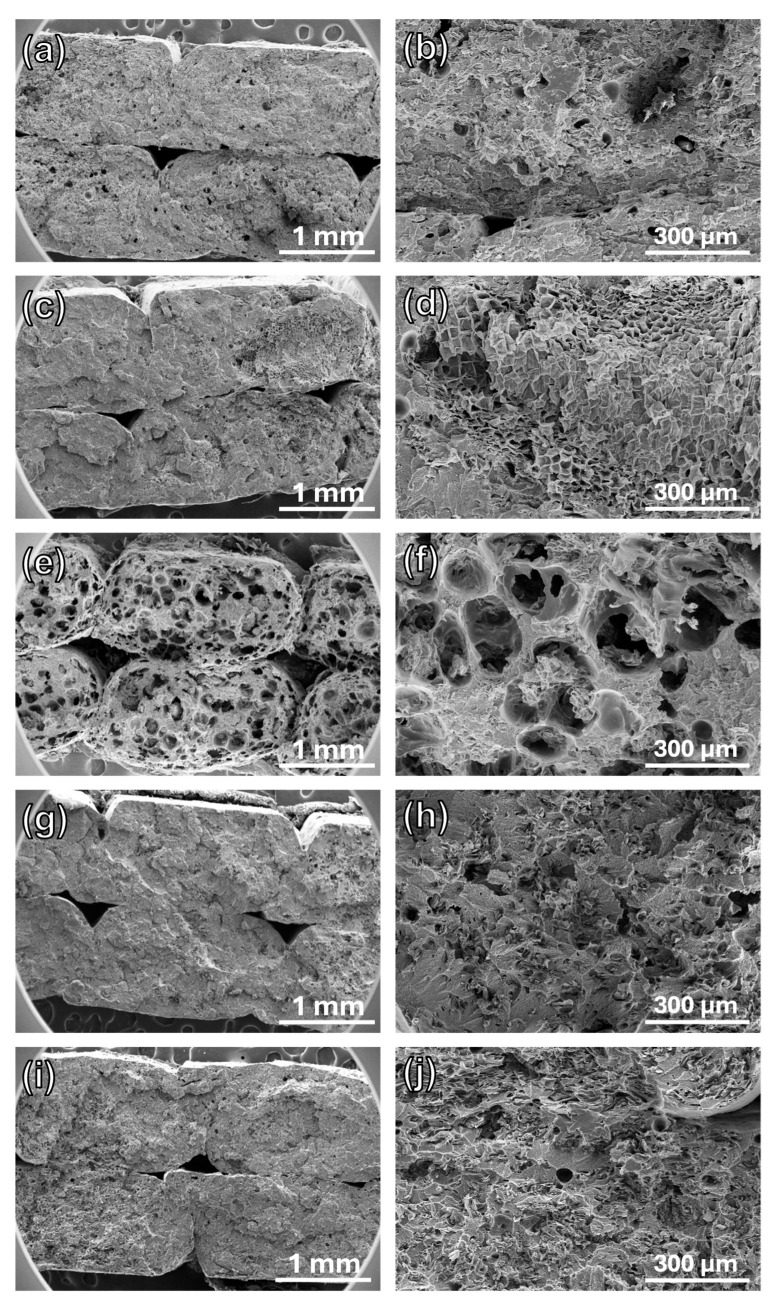
Scanning electron micrographs of the fracture surface of XY tensile specimens of the (**a**,**b**) 11FC_15, (**c**,**d**) 11CC_15, (**e**,**f**) 14FC_15, (**g**,**h**) 14FC_5 and (**i**,**j**) 14FC10 cork composites.

To corroborate these results, the fracture surfaces of the XY specimens of composites 11FC_15, 11CC_15, 14FC_15, 14FC_5, and 14FC_10 were analyzed using SEM. Figure 7 shows flat fracture, characteristic of brittle fracture, for all cases. This means that the cork participated in the fracture mechanism, embrittling the ASA matrix, as also observed for the mechanical properties, as the elongation at break values decreased with the presence of either FC or CC. The SEM images evidence the presence of the cork’s cellular structure, although this structure mostly collapsed in almost all cases, which explains why the objects printed using FGF had a density practically identical to that of pure ASA. In the case of 11CC_15, Figure 7d shows that the internal part of the cork particle can keep its cellular structure. It seems that these particles, being larger, preferentially collapse at their outer part, allowing their internal structure to remain unaltered. In other words, larger particle sizes potentially result in cork composites without their structure collapsing. However, as previously observed, this does not represent a significant improvement in mechanical or physical properties, so further research is needed. However, the fracture surface of 14FC_15 (Figure 7e,f) showed a highly porous surface, with pores much larger than those characteristic of cork cells, in the order of hundreds of microns. The presence of uncrushed cork particles, which kept their cellular structure, can also be observed within these large pores. This supports our previous hypothesis that cork particles release gases during their partial degradation under the influence of printing via FGF, resulting in a foam-like porous structure, which simultaneously protects the cork particles from collapse. Interestingly, this effect was only clearly visible in 14FC_15, when the concentration of cork particles was higher. SEM images of 14FC_5 and 14FC_10 (Figure 7g–j) show certain roughness and porosity outcomes, which may have been due to the smaller amount of gas released as a result of the cork’s degradation. However, it seems that in these composites, the amount of cork did not reach a critical threshold for the formation of gas bubbles that would have given rise to a porous structure such as that observed in 14FC_15.

**Figure 8:**
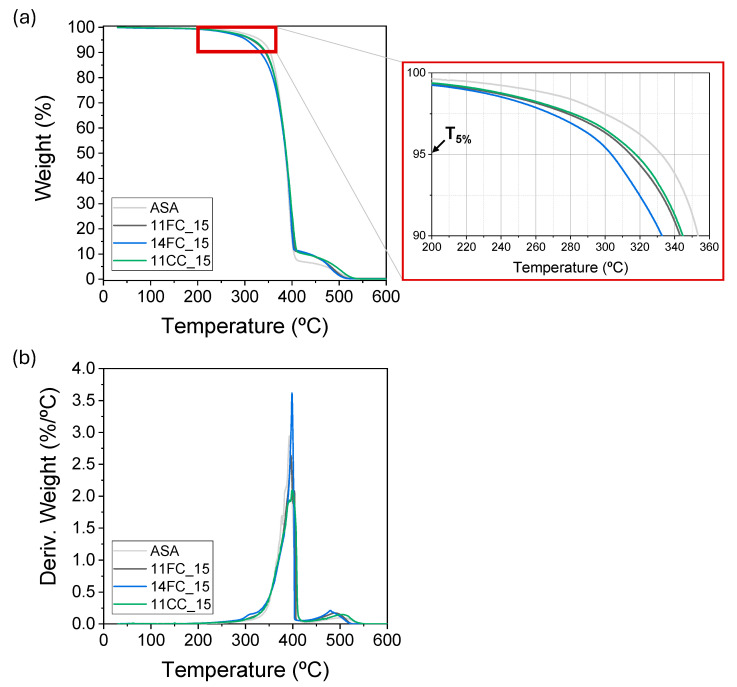
(**a**) TGA and (**b**) DTG curves of ASA, 11FC_15, 14FC_15 and 11CC_15 composites.

The thermal properties of the composites with 15 wt.% cork previously observed via SEM were analyzed. Figure 8 shows the thermal degradation curves obtained using TGA, from which we can see how, in all cases, the thermal stability of the composites decreased slightly with respect to that of pure ASA. In particular, the degradation temperatures of these materials, calculated as the temperature at which their initial mass decreased by 5% (T_5%_), are 332, 314, 316 and 302 °C for ASA, 11FC_15, 11CC_15 and 14FC_15, respectively. This also demonstrates that the cork composites prepared with 14FC also had lower thermal resistance, probably because, in this case, the cork had not yet been completely degraded (i.e., carbonized) in the manufacturing process, making it less thermally stable [44,45]. In the cases of 11FC and 11CC, the cork had already been previously degraded and transformed (at least partially), which would have contributed to greater thermal stability than that of undegraded cork. The maximum degradation speed, observed in the DTG curve, however, shows minimal differences, with values between 393 and 398 °C for all cases, evidencing that the cork mostly impacted the beginning of the degradation of the material.

**Figure 9:**
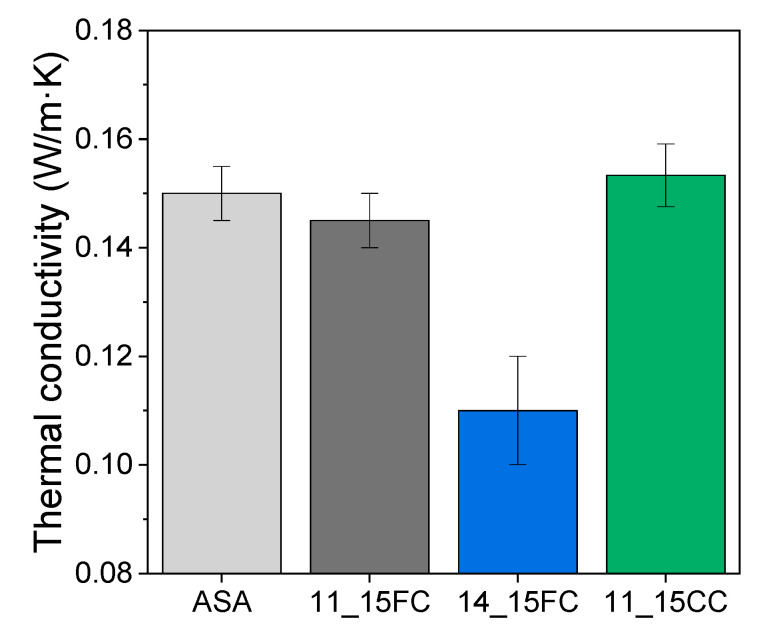
Thermal conductivity values of ASA, 11FC_15, 14FC_15 and 11CC_15 composites.

Finally, the thermal conductivity of ASA, 11FC_15, 11CC_15, and 14FC_15 was also measured to study the influence of cork as a thermal insulator in these composites (Figure 9). 11FC_15 and 11CC_15 did not present statistically significant differences with respect to pure ASA. Although it has been previously observed that some cork particles can keep their cellular structure after printing (particularly in the case of 11CC_15), the voids generated during FGF between layers must be considered, as these also contribute to a decrease in the thermal conductivity of the material in general. Therefore, it seems that in these cases, the contribution of the cork particles was not large enough to observe an improvement in the thermal insulation of these composites. 14FC_15, however, showed a decrease of more than 25% in thermal conductivity, mainly due to the generation of large pores by the FC particles during the FGF process. This, as previously observed in the SEM images in Figure 7, allowed for the printing of a rigid foam-like polymer structure with enhanced thermal properties as an insulator, while presenting acceptable mechanical properties compared to other cork composites [24,29]. It should be noted that this composite could only be manufactured with CF, so in this case, particle size has proven to be a critical element in the design of the feedstock material.

## 4. Conclusions

In this work, a series of cork composites were developed using different particle sizes (FC, Dp < 250 µm and CC, 1 mm < Dp < 2 mm) and varying compounding processing conditions to study their influence on the final properties of objects manufactured by FGF using a LFAM printer. In particular, different inlets within the twin-screw extruder were tested, and it was observed that ASA composites with up to 15 wt.% of CC and 20 wt.% of FC could be processed. Furthermore, introducing cork powder at the last inlet of the extruder (i.e., inlet 4, closest to the nozzle) reduced its residence time during compounding, minimizing degradation and resulting in pellets of a lighter color. This color remained largely unchanged after FGF printing, and all composites could be adequately printed under the same optimized operating conditions that minimized cork degradation. A linear decrease in the mechanical properties of all composites was observed as the cork content increased. While some minor differences were observed between samples (e.g., 11CC_15 partially retained the cork structure), statistical analysis (ANOVA) indicated that particle size has a limited effect on the mechanical, physical (i.e., density), and thermal properties of the composites under the studied processing conditions. Composite 14FC_15 partially degraded during printing, forming a highly porous, foam-like material with reduced density and thermal conductivity. This effect did not occur for cork amounts below 15 wt.%, suggesting that a minimum concentration of cork is required to generate this foam-like structure.

Overall, this work demonstrates the potential for cork waste valorization to develop more sustainable composites, reducing the amount of petroleum-derived plastic used. The results suggest that the influence of cork particle size is minor under the processing conditions studied, indicating that steps such as grinding and sieving could potentially be simplified, thus streamlining manufacturing. Finally, this study shows that polymer foams can be generated in situ during FGF printing, enabling the fabrication of lightweight and insulating objects.

## Figures and Tables

**Table 1:** List of composites tested in this work using FC.

Sample Name	Inlet ASA	Inlet FC	Cork Concentration (wt.%)	Successful Extrusion
**11FC_5**	1	1	5	Yes
**11FC_10**	1	1	10	Yes
**11FC_15**	1	1	15	Yes
**11FC_20**	1	1	20	Yes
**11FC_25**	1	1	25	No
**22FC_5**	2	2	5	Yes
**22FC_10**	2	2	10	Yes
**22FC_15**	2	2	15	Yes
**22FC_20**	2	2	20	No
**33FC_5**	3	3	5	No
**44FC_5**	4	4	5	No
**14FC_5**	1	4	5	Yes
**14FC_10**	1	4	10	Yes
**14FC_15**	1	4	15	Yes
**14FC_20**	1	4	20	No

**Table 2:** List of composites tested in this work using CC.

Sample Name	Inlet ASA	Inlet CC	Cork Concentration (wt.%)	Successful Extrusion
**11CC_5**	1	1	5	Yes
**11CC_10**	1	1	10	Yes
**11CC_15**	1	1	15	Yes
**11CC_20**	1	1	20	No
**22CC_5**	2	2	5	Yes
**22CC_10**	2	2	10	Yes
**22CC_15**	2	2	15	Yes
**22CC_20**	2	2	20	No
**33CC_5**	3	3	5	No
**44CC_5**	4	4	5	No
**12CC_5**	1	2	5	No
**14CC_5**	1	4	5	No

## Data Availability

Data are contained within the article. Raw data will be made available by the authors upon reasonable request.
